# Synthesis of Pure Al and Al-GNP Composites via Powder Metallurgy for the Subsequent Development of Nanostructured Thin Films Using PLD

**DOI:** 10.3390/molecules31101711

**Published:** 2026-05-18

**Authors:** Rosalba Castañeda-Guzmán, Roberto Ademar Rodríguez-Díaz, Rafael Felix-Contreras, Jesús Armando Lucero-Acuña, Jonathan de la Vega Olivas, Paul Zavala-Rivera, Jesús Porcayo-Calderon

**Affiliations:** 1 Instituto de Ciencias Aplicadas y Tecnología, UNAM, Circuito Exterior S/N, Ciudad Universitaria, Alcaldía Coyoacán, Ciudad de Mexico 04510, Mexico; rosalba.castaneda@icat.unam.mx; 2 Ingeniería Mecatrónica, Tecnologico de Estudios Superiores de Tianguistenco, Km. 22, Carretera Tenango—La Marquesa, Poblado de Santiago Tilapa, Santiago Tianguistenco 52650, Mexico; 3 Departamento de Ingenieria Quimica y Metalurgia, Universidad de Sonora, Hermosillo 83000, Mexico; a223230118@unison.mx (R.F.-C.); armando.lucero@unison.mx (J.A.L.-A.); jonathan.delavega@unison.mx (J.d.l.V.O.); paul.zavala@unison.mx (P.Z.-R.)

**Keywords:** graphene nanoplatelets, recycled aluminum, pulsed laser deposition, metal matrix composites, nanoscience, green chemistry, microstructural evolution, thin films

## Abstract

While aluminum (Al) continues to be a cornerstone for microelectronic interconnect technologies, its chronic tendency toward hillock growth and thermal instability necessitates a transition toward high-performance nanostructured material architectures. This research tackles these reliability bottlenecks by achieving a molecular-level integration of graphene nanoplatelets (GNPs) within Al matrices, a strategy designed to fortify structural resilience. Adopting a green chemistry approach, we synthesized Al-GNP (0.25 vol.%) composite thin films through Pulsed Laser Deposition (PLD) using precursors derived from recycled aluminum. A major obstacle—the formation of the deleterious Al_4_C_3_ intermetallic phase—was effectively suppressed by ensuring a homogeneous supramolecular dispersion via a specialized dual protocol (ultrasonication and magnetic stirring) during the powder metallurgy stage. Comprehensive physicochemical characterization, utilizing HR-TEM and XRD, verified the structural integrity of the multilayer GNPs (d-spacing = 4.6 Å). Furthermore, surface metrology analysis uncovered a radical shift in growth kinetics: whereas pure Al grew via a “spiky” Volmer-Weber mechanism (Sku = 31.17), the carbon-based inclusion stabilized the film evolution, tempering the kurtosis to Sku = 7.74. Analytical cross-sectional EDS confirmed both stoichiometric fidelity and the achievement of void-free Si/Pt/Al-GNP interfaces. These outcomes prove that a precise nanoscale tailoring of surface morphology via carbonaceous reinforcements significantly bolsters microstructural stamina. Consequently, these PLD-deposited composites emerge as sustainable, cutting-edge candidates for the next generation of microelectronic packaging and interfacial chemistry applications.

## 1. Introduction

Since the emergence of the first integrated circuits in the 1960s, our ability to miniaturize technology has grown at a staggering pace, transforming nearly every aspect of modern life. At the heart of this evolution lies aluminum (Al), a material that has become indispensable due to its excellent conductivity and cost-effectiveness. However, as we strive to manufacture increasingly dense and powerful devices, aluminum has begun to reach its physical limits. The challenge is not only its electrical resistance but also its behavior under thermal stress: the formation of tiny protrusions known as “hillocks” during processing can ruin an entire chip in an instant [1,2]. Recent studies in X-ray diffraction and operando techniques have highlighted how microstructural evolution in Al-alloys under thermal aging remains a critical bottleneck for reliability in high-performance applications [3,4].

In the face of this challenge, materials science has sought innovative solutions. One of the most promising approaches does not come from another metal, but from carbon-based nanomaterials such as graphene nanoplatelets (GNPs). These materials, derived from graphitic carbon, exhibit a highly ordered sp^2^-bonded structure and remarkable mechanical and functional properties. This two-dimensional material has captivated the scientific community due to its exceptional mechanical properties, including an intrinsic tensile strength of approximately 130 GPa and a Young’s modulus near 1 TPa, as well as an outstanding in-plane thermal conductivity in the range of ~2000–5000 W·m^−1^·K^−1^. [5]. By integrating these tiny graphene sheets into an aluminum matrix, we are not just seeking a stronger metal, but a composite material capable of withstanding the demands of next-generation electronics, potentially enhancing properties like field emission and electromagnetic interference shielding [6,7].

Yet, producing this “perfect material” remains challenging. Graphene tends to agglomerate due to van der Waals interactions, while processes such as mechanical milling can damage its lattice, introducing defects detectable by Raman and SEM [8]. These imperfections hinder phonon transport, lowering thermal conductivity, and act as stress concentrators, reducing mechanical strength. Therefore, maintaining graphene’s structural integrity during processing is essential to preserve its properties. Therefore, in this work, we have chosen a more careful path: a combination of ultrasonic dispersion and magnetic stirring that allows the graphene to unfold naturally and homogeneously within the aluminum. This liquid-phase exfoliation approach has been proven effective for maintaining the quality and thickness control of graphene during large-scale production [9]. Through Powder Processing, we transform these mixtures into solid bulk materials that serve as the foundation of our research.

The ultimate goal of this study is to bridge the gap between bulk materials and thin-film technology. Using the Pulsed Laser Deposition (PLD) technique, we have succeeded in “projecting” the exact composition of our composite materials onto high-precision silicon substrates. PLD is currently regarded as one of the most versatile methods for achieving large-scale, high-precision thin films with complex stoichiometries [10]. This approach not only allows us to study how graphene-reinforced aluminum behaves at a microscopic scale—overcoming the limitations of traditional magnetron sputtering for blooming microstructures [11]—but also opens the door to new ways of manufacturing electronic components that are more stable, durable, and efficient.

## 2. Materials and Methods

### 2.1. Pre-Treatment and Melting of Recycled Aluminum

Recycled aluminum was cleaned by manually sanding the cans with solvents to remove paint while preserving material integrity. Subsequently, the units were washed with cleaning agents to eliminate internal residues, then rinsed and dried for further processing. The melting process was conducted at 950 °C to ensure optimal fluidity and compensate for thermal losses. Fluxing, deoxidizing (using magnesium), and degassing agents were incorporated into the molten bath to remove slag, prevent oxide formation, and minimize hydrogen-induced porosity. This purification stage is critical when working with recycled sources to preserve mechanical properties, as the reuse of metallic powders and scrap often requires strict control over oxide inclusions to ensure consolidation integrity [12,13].

Table 1 provides the typical chemical composition of aluminum commonly used in beverage cans, based on literature reports. Beverage can alloys are generally Al–Mn–Mg-based systems containing minor amounts of Fe, Si, Cu, and Zn, depending on recycling stream and manufacturing source. This reference composition offers a reasonable estimation of the expected impurity range in the recycled aluminum feedstock employed in this work.

### 2.2. Powder Production and Granulometric Classification

Recycled aluminum powder was produced via mechanical comminution using high-purity hardened steel files on previously cast ingots. The resulting particles underwent granulometric classification using #200 (75 μm) and #325 (45 µm) mesh sieves to ensure a controlled particle size distribution. Precise control of particle size is essential for subsequent consolidation stages, as it directly influences the packing density and the surface area available for reinforcement bonding [14]. To support particle size control after sieving, a granulometric analysis was conducted. The distribution spans approximately 5 µm to 45 µm, with nearly 70% of particles below 30 µm and a median size (D50) around 25–27 µm. The retained fraction peaks in the intermediate range, indicating a balanced distribution of fine and coarse particles. This combination enhances packing efficiency and structural stability during compaction, favoring improved densification and interfacial bonding in subsequent processing stages.

### 2.3. Dispersion and Synthesis of Al-GNP Mixtures

The graphene nanoplatelets (GNPs) used in this study were supplied as a black powder by Sigma-Aldrich (St. Louis, MO, USA), with a true density of 2.0–2.25 g/cm^3^ and a bulk density of 0.2–0.4 g/cm^3^. They exhibit a high specific surface area (~500 m^2^/g), a lateral size below 2 μm, and a thickness of a few nanometers, consistent with a nanoscale layered structure. The material also presents a high purity level (quality grade 100).

Once the Al–GNP mixtures were prepared by establishing a concentration of 0.25 vol.% GNP (in order to prevent the formation of deleterious Al_4_C_3_ carbide, which can induce embrittlement in the composite), the mixtures were dispersed in 100 mL of ethanol (99% purity). Subsequently, the samples underwent an ultrasonication process at a frequency of 25 kHz for 1 h. Following the ultrasonic treatment, the suspension was subjected to magnetic stirring at room temperature for 1 h at 1150 RPM to ensure the homogeneity of the mixture. This dual-dispersion approach—ultrasonication followed by stirring—has been shown to effectively minimize the agglomeration of graphene nanoplatelets without inducing the structural damage typically associated with high-energy ball milling [9]. Following dispersion, the Al-GNP ethanol suspensions were dried in an oven at 90 °C for 8 h to ensure total evaporation of the solvent.

### 2.4. Compaction and Sintering

High-purity Al and Al-GNP mixtures were uniaxially pressed at 600 MPa for 3 min to obtain green compacts. Sintering was conducted in a muffle furnace at 500 °C for 4 h to promote diffusion bonding. To prevent oxidation, specimens were encapsulated in a stainless-steel box within a refractory brick powder bed. Such protective environments during thermal cycles are vital for aluminum composites, where the presence of oxygen can lead to the formation of detrimental Al_2_O_3_ interfaces that hinder load transfer between the matrix and the reinforcement [6].

### 2.5. Thin-Film Deposition by PLD

Al-based films were deposited onto Pt-coated silicon substrates by Pulsed Laser Deposition (PLD). An Nd:YAG laser (λ = 1064 nm, τ = 10 ns) was operated at a fluence of approximately 6 J/cm^2^, a repetition rate of 10 Hz, and a base pressure of 5 × 10^−6^ Torr. The deposition process was performed for 1.5 h at room temperature. The use of PLD ensures the stoichiometric transfer of the composite target to the substrate, a key advantage over other PVD techniques when maintaining the precise Al-GNP ratio in thin-film architectures for microelectronic applications [10].

### 2.6. Characterization Techniques

#### 2.6.1. Electron Microscopy and Chemical Analysis (FE-SEM & EDS)

The morphology of the raw Graphene Nanoplatelets (GNPs) was characterized using a Field Emission Scanning Electron Microscope (FE-SEM, JSM-7401F, JEOL Ltd., Tokyo, Japan)equipped with an Energy-Dispersive X-ray Spectroscopy (EDS) detector. This system was employed to obtain high-resolution micrographs of the GNPs and to perform EDS line-scan chemical analyses. Recent advances in tandem XPS-SEM/EDS analysis have demonstrated that this combination is essential for accurately determining the elemental composition and functionalization state of graphene nanoplatelets, ensuring that no significant impurities are introduced during processing [15]. Additionally, FE-SEM was utilized to investigate the surface and cross-sectional microstructure of the Al and Al-GNP thin films deposited on Pt-coated Si substrates. X-ray elemental mappings were also acquired to evaluate the chemical distribution across the surface and transverse sections of the films, a critical step for verifying the homogeneous incorporation of carbonaceous phases within metallic matrices [8].

#### 2.6.2. High-Resolution Structural Assessment (HR-TEM)

Detailed structural analysis of the GNPs was conducted via High-Resolution Transmission Electron Microscopy (HR-TEM, JEM-2200FS, JEOL Ltd., Tokyo, Japan). This technique allowed for the measurement of interplanar distances of the predominant crystalline planes and the evaluation of the thickness of the stacked nanoplatelet agglomerates. In situ TEM observations are currently considered the gold standard for assessing the structural stability and atomic arrangements of carbon nanomaterials, providing a direct visualization of the lattice integrity that other techniques cannot resolve [16].

#### 2.6.3. X-Ray Diffraction (XRD) Analysis

The crystalline structure of the Al and GNP powders was analyzed by X-ray Diffraction (XRD) using a Panalytical Philips X’Pert diffractometer (PANalytical B.V., Almelo, The Netherlands) with CuK_α_ radiation (λ = 1.5406 Å). The scanning range was set from 20° to 70° (2θ°) with a step size of 0.017°. For the pure Al ingot and the consolidated Al-0.25 vol.% GNP specimens, the XRD analysis was extended to a range of 20° to 120° (2θ°) to capture higher-order reflections. Accurate XRD peak broadening modeling is vital for Al-alloy characterization, as it allows for the differentiation between crystallite size effects and microstrain induced by the presence of GNPs in the matrix [17].

#### 2.6.4. Optical Microscopy and Chemical Etching

Finally, the microstructural evolution of the consolidated samples was examined using a Zeiss Axio optical microscope (Carl Zeiss AG, Oberkochen, Germany). To reveal the grain boundaries and secondary phases, the surface of the sintered pellets was chemically etched with Keller’s reagent (2% HF, 3% HCl, 5% HNO_3_, and 90% H_2_O) prior to observation. This traditional metallographic approach remains indispensable for evaluating the grain refinement and the effectiveness of the sintering process in aluminum-based composites prepared by Particulate processing [18].

#### 2.6.5. Atomic Force Microscopy Characterization

Atomic force microscopy (AFM) was performed in tapping mode under ambient conditions using a silicon probe (tip radius < 10 nm). Surface scans (1–5 µm) were acquired at ≥512 × 512 resolution and a scan rate of ~0.5–1 Hz to capture pronounced topographical features. The vertical range enabled characterization of submicron height variations. Areal roughness parameters (Sa, Sq, Ssk, Sku) and line profiles were obtained from the topographic data.

#### 2.6.6. Microhardness Testing

Vickers microhardness measurements were performed using an LM300AT microhardness tester (LECO Corporation, St. Joseph, MI, USA). Prior to testing, the sintered pellet surfaces were carefully polished to obtain a mirror-like finish. A load of 100 g was applied, and eight indentations were carried out on each specimen to ensure reproducibility.

## 3. Results and Discussion

### 3.1. Microstructural Characterization During Synthesis of Al-Based Targets

#### 3.1.1. Microstructural Characterization via X-Ray Diffraction of the Starting Materials (Powder and Consolidated Forms)

**Figure 1:**
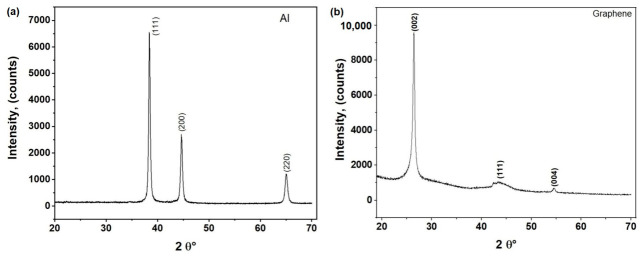
XRD patterns of the starting materials: (**a**) recycled Al powder obtained from the melting and casting of used beverage cans (UBCs), and (**b**) as-received graphene nanoplatelets (GNPs) in powder form.

As illustrated in the XRD patterns of Figure 1a, the aluminum powder derived from recycled beverage cans exhibits sharp, well-defined reflections synonymous with the face-centered cubic (FCC) lattice of α-Al. This spectral clarity confirms that the structural integrity and high crystallinity of the metal remain uncompromised after the pulverization stage. Notably, the absence of detectable secondary phases—such as alumina (Al_2_O_3_) or undesired intermetallics—is consistent with established benchmarks for high-purity powders in powder technology frameworks. The dominance of clean FCC reflections at 2θ = 38.5°, 44.7°, and 65.1° (corresponding to the (111), (200), and (220) planes, respectively) further underscores that the recycling and milling protocols effectively mitigated oxidation and cross-contamination [12,14].

The XRD profile of the as-received GNP powder, depicted in Figure 1b, reveals prominent reflections at the (002), (101), and (004) planes, which are hallmarks of the hexagonal graphite lattice (P6_3_/mmc). The presence of these sharp peaks validates the structural integrity and long-range order of the graphene nanoplatelets used in this study. Notably, the (002) diffraction peak at 2θ = 26.4° corresponds to an interplanar d-spacing of approximately 0.34 nm, a value characteristic of well-stacked, non-oxidized graphene layers. This observation is crucial as it distinguishes the precursor from graphene oxide (GO), which typically exhibits a significant lattice expansion (d = 0.8 nm) and a resultant peak shift toward lower angles (~10–11°) due to the intercalation of oxygen-containing functional groups [19,20,21].

**Figure 2:**
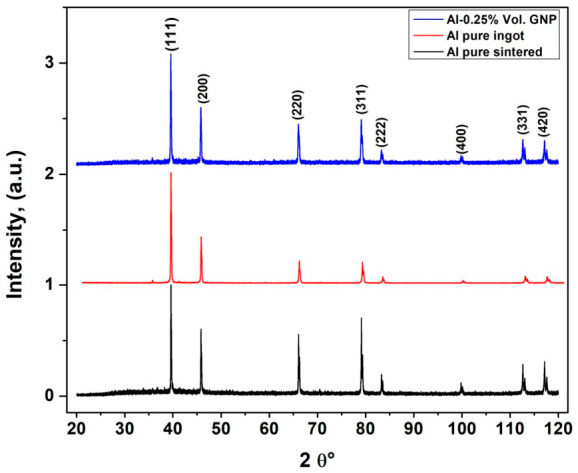
Comparative X-ray diffraction analysis of pure Al (ingot and sintered) and consolidated Al-GNP (0.25 vol.%) composite fabricated from recycled aluminum cans via solid-state processing route.

Figure 2 shows the normalized XRD patterns of the Al–0.25 vol.% GNP composite and pure aluminum samples (ingot and sintered), using the Al (111) peak as a reference. All profiles exhibit the characteristic reflections of face-centered cubic (FCC) aluminum: (111), (200), (220), (311), (222), (400), (331), and (420), confirming preservation of the Al matrix after processing and GNP addition. After normalization and peak alignment correction, the reflections of the three samples appear at comparable 2θ positions, indicating that the previously observed minor shifts were likely caused by specimen positioning rather than lattice changes. No additional peaks related to oxides, contaminants, or secondary phases were detected. In particular, no clear Al_4_C_3_ reflections were observed, although trace amounts below the XRD detection limit cannot be excluded. Overall, the similarity of the profiles indicates that 0.25 vol.% GNP did not significantly modify the bulk crystal structure of aluminum, while phase integrity was maintained during consolidation [17,22].

As evidenced in Figure 2, the XRD profiles of the pure ingot and the sintered aluminum specimens exhibit indistinguishable characteristic reflections, both manifesting high-intensity FCC peaks without detectable contaminants. This spectral congruence confirms that the thermal cycles inherent to melting, casting, and solid-state sintering effectively preserved the phase purity of the matrix, mirroring observations reported for advanced Al-Cu and Al-Sc-Zr systems. Such consistency underscores the capacity of the powder fabrication method to replicate the crystalline architecture of bulk-cast ingots while circumventing deleterious casting defects, such as sludge inclusions or shrinkage porosity. Furthermore, the absence of extraneous peaks corroborates those interfacial reactions and oxidation remained negligible during consolidation—a finding that aligns with the attainment of relative densities exceeding 95% in oxide-passivated Al powders [3,17].

The absence of detectable Al_4_C_3_ peaks may suggest limited interfacial reactions between GNPs and the aluminum matrix during sintering; however, this interpretation should be considered with caution due to the low GNP concentration and the corresponding detection limits of XRD. Therefore, the discussion is complemented with microstructural observations, which provide additional insight into the interfacial characteristics beyond the sensitivity of XRD analysis. In the end, this sintered composite holds phase quality on par with the ingot, opening doors to better mechanical strength without the uneven phases you often get in cast parts. Down the line, digging into XRD peak broadening could spotlight any leftover stresses or grain refining from the GNPs [4,18,22].

#### 3.1.2. Microstructural Characterization via SEM of Graphene Nanoplatelets

**Figure 3:**
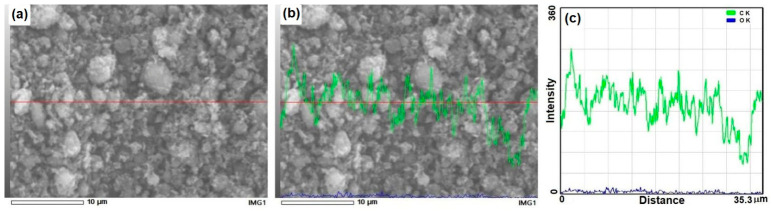
(**a**,**b**) SE-SEM micrographs of the GNP powders showing the horizontal red line used for the scan-line chemical analysis and (**c**) scanline chemical analysis plot of SE-SEM micrograph c.

The SE-SEM micrograph in Figure 3c shows an EDS elemental line scan of ~35.29 µm, illustrating the distribution of C and O across the graphene nanoplatelets (GNPs). The carbon signal intensity dominates and exhibits stable oscillations around a mean value, while the oxygen signal remains negligible, close to the baseline, indicating the absence of oxygen-rich functional groups and confirming no graphene oxide phase was formed, thus verifying the high purity of the carbonaceous reinforcement [15,23].

These results align with studies from the last seven years on GNPs in powder form, where SEM/EDS reveals low O/C ratios (<0.05) in electrochemically exfoliated or high-purity commercial materials, contrasting with graphene oxides that exhibit intense O peaks. Reviews of SEM imaging highlight that stable C oscillations, as observed here, reflect uniform exfoliation and lateral sizes of 5–15 µm without oxidative agglomerates, similar to GNPs in LDPE nanocomposites where concentrations >2.5% induce inhomogeneities absent in this case. The 35 µm linear profile exceeds typical scan line resolutions (e.g., 90 pA in fluorinated GNPs), validating elemental homogeneity without O gradients [8,15,23,24].

#### 3.1.3. Microstructural Characterization via HR-TEM of Graphene Nanoplatelets

**Figure 4:**
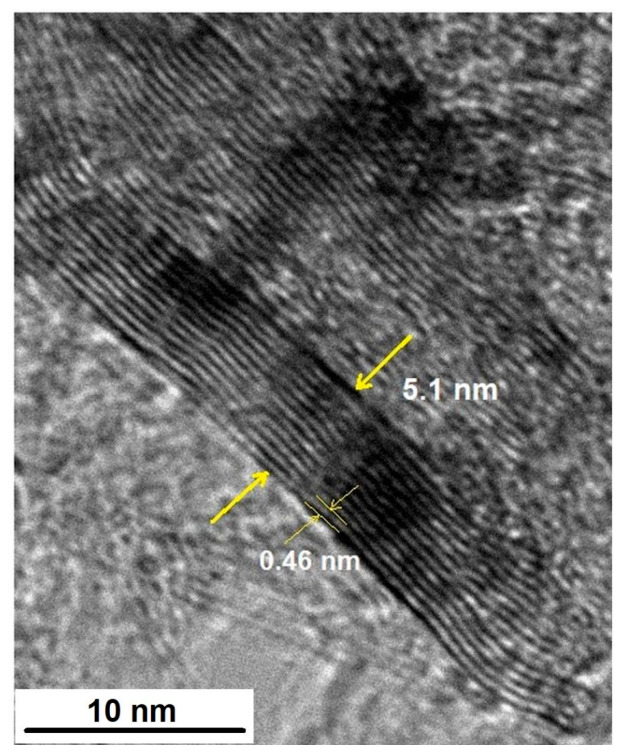
HR-TEM Micrograph of GNPs. A subset of graphene nanoplatelets is highlighted with yellow arrows.

Figure 4 from the HR-TEM image clearly reveals the lattice fringes corresponding to the (002) planes of graphene, with a measured d-spacing of 4.6 Å, which is significantly larger (~37%) than the typical value for graphitic structures (~3.35 Å). This pronounced increase suggests substantial interlayer expansion and/or structural disorder within the nanosheets, rather than a minor deviation. Nearby, a GNP stack measures 5.1 nm thick, corresponding to approximately 11–12 atomic layers (Figure 4), consistent with multilayer GNPs (2–10 nm) produced via exfoliation or milling [5,7].

Such expanded d-spacing aligns with prior HR-TEM observations of custom GNPs, capturing irregular stacking and synthesis-induced defects that yield flakes of 0.2–25 μm for effective mechanical integration in composites. The layer count matches water-dispersed GNPs, featuring thin transparent edges alongside thicker dark stacks, with TEM typically underestimating lateral dimensions relative to DLS. Raman-HRTEM correlation confirms successful exfoliation without structural damage, as seen in GNP-polymer systems [9].

This subtle expansion preserves sp^2^ integrity while introducing beneficial edge disorder, akin to in situ HR-TEM of irradiated carbon structures where defects migrate internally without amorphization. In PLA/GNP or Al-matrix nanocomposites, it enables EMI shielding and thermal conductivity (~1.72 W/m·K at 15 wt%) without excessive delamination. HR-TEM grain mapping here surpasses TKD for accurate layer quantification, enabling optimized GNP loadings while maintaining matrix uniformity [16,25].

### 3.2. Morphological and Compositional Analysis of Al–GNP Powders

**Figure 5:**
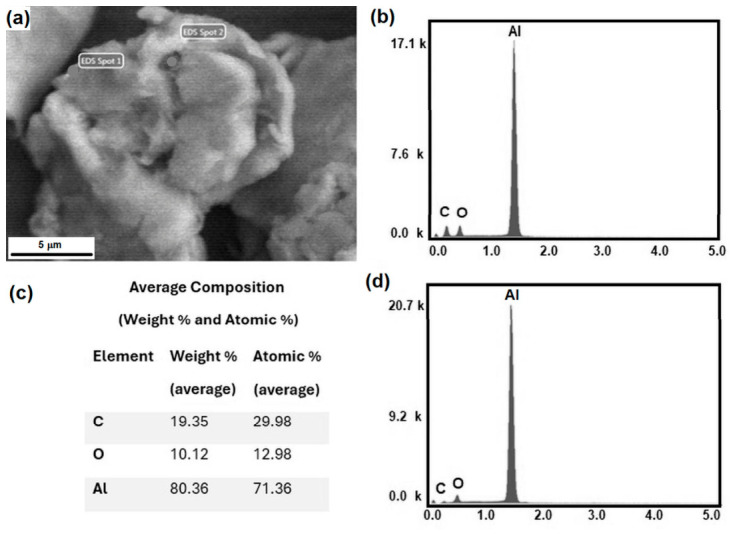
(**a**) Scanning electron micrograph of the Al–0.25 vol.% GNP mixture; (**b**,**d**) energy-dispersive spectroscopy (EDS) spectra obtained from two selected regions (Spot 1 and Spot 2); and (**c**) average compositions derived from semiquantitative chemical analyses.

Figure 5a presents a scanning electron micrograph of the Al–0.25 at.% GNP powder mixture, where aluminum particles are predominantly observed. The presence of graphene nanoplatelets is difficult to distinguish, which can be attributed to their low concentration within the mixture. Nevertheless, EDS analyses were conducted at locations where small dark features were identified under higher magnification.

As shown in Figure 5a, two specific regions on a single aluminum particle were selected and labeled as EDS spot 1 and EDS spot 2, where localized chemical analyses were performed. The corresponding EDS spectra obtained from these regions are presented in Figure 5b,d. Both spectra confirm the presence of Al, O, and C. Additionally, Figure 5c displays the results of the semi-quantitative analysis in terms of atomic percentage (at.%) and weight percentage (wt.%).

#### TEM Analysis of Al–GNP Powder Mixtures

In order to analyze the dispersion of graphene nanoplatelets (GNPs) on aluminum particles in the Al–0.25 vol.% GNP powder mixture, transmission electron microscopy (TEM) was employed. Similar to the observations obtained by SEM, the identification of GNPs within the powder mixture remained challenging, which can be attributed to their low concentration. Nevertheless, selected regions of the sample were examined in detail.

**Figure 6:**
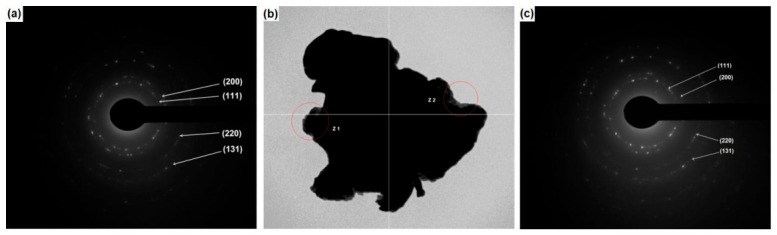
(**a**,**c**) Selected-area electron diffraction (SAED) patterns of the Al–0.25 vol.% GNP composite obtained from the two regions, Z1 and Z2, indicated in (**b**), which shows the transmission electron micrograph of a particle.

Figure 6b shows two electron-transparent regions within a particle, labeled Z1 and Z2. These areas were analyzed using selected area electron diffraction (SAED). The resulting diffraction patterns exhibit characteristic rings with discrete spots along their circumference, indicating the nanostructured nature of the material. According to the diffraction patterns, these features correspond to the face-centered cubic (fcc) crystal structure of aluminum.

Figure 6a presents the indexed diffraction rings associated with the (111), (200), (220), and (311) crystallographic planes, while Figure 6c shows similar ring patterns with the same Miller indices. In both cases, the observed diffraction features are consistent with the fcc structure of aluminum.

- Microstructural Characterization of Al Thin Films Deposited on Pt-Coated Silicon Substrates

**Figure 7:**
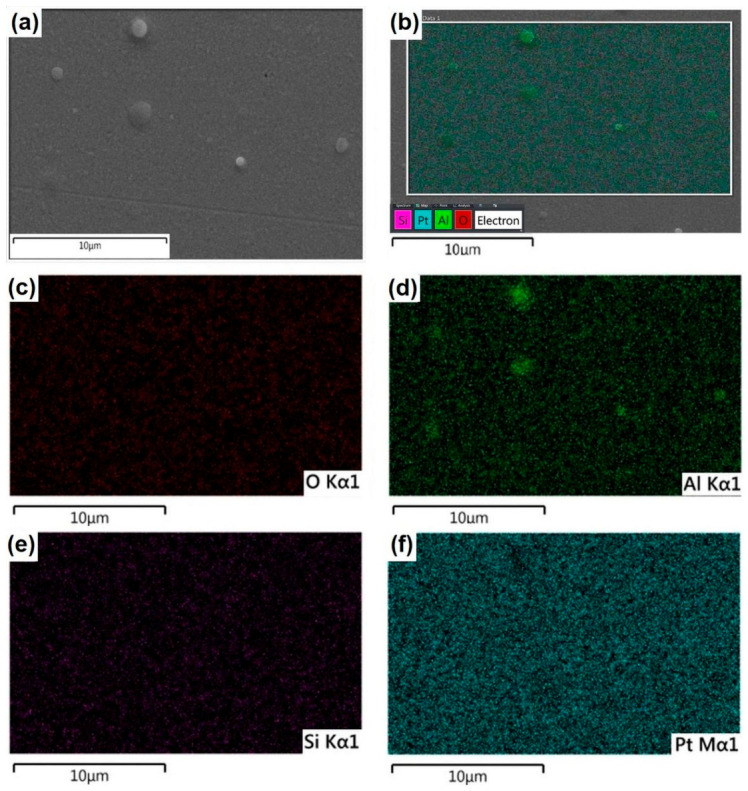
Surface characterization of the Al thin film deposited on a Pt-coated Si substrate. (**a**) SEM micrograph showing the surface morphology of the pure Al layer synthesized by PLD. (**b**) Combined EDS elemental map illustrating the spatial distribution of all detected elements within the area shown in (**a**). (**c**–**f**) Individual EDS elemental maps showing the distribution of (**c**) O, (**d**) Al, (**e**) Si, and (**f**) Pt, respectively.

Figure 7a displays the surface microstructure of the Al layer, which uniformly covers the underlying substrate. The PLD process promoted the formation of spherical nano- and micro-particles, clearly visible across the film’s morphology. The combined elemental map in Figure 7b reveals that the Al signal (magenta) is predominantly distributed across the entire scanned area. Within this region, the detection of O is attributed to the spontaneous formation of a passive Al_2_O_3_ layer on the aluminum surface. Furthermore, signals from Pt and Si (Figure 7e,f) were also detected within the Al-mapped area. This is a direct consequence of the electron beam interaction volume; this pear-shaped region extends from the surface to a depth of approximately 5 μm within the specimen, acting as the emission source for secondary electrons, backscattered electrons, and characteristic X-rays. Since this interaction depth exceeds the combined thickness of the Al film and the Pt interlayer, the underlying substrate signals are inevitably captured. Consequently, Figure 7e,f confirm that the Si signal is concentrated in the substrate, consistent with the layered architecture of the sample.

**Figure 8:**
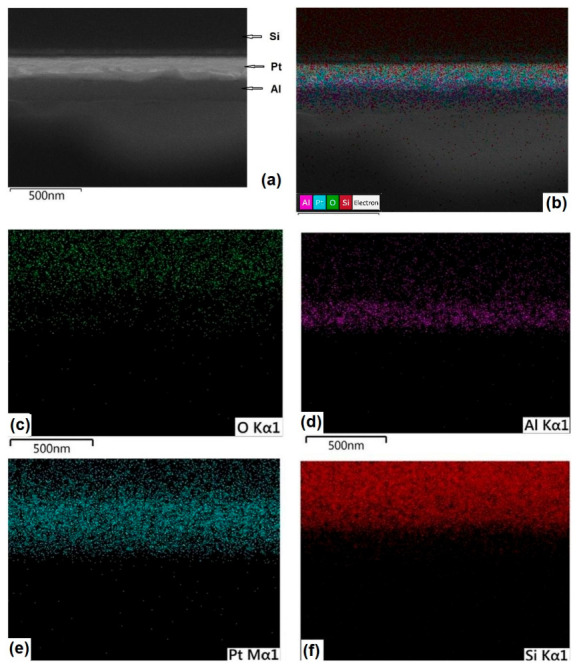
Cross-sectional characterization of the Al thin film deposited on a Pt-coated Si substrate. (**a**) SEM micrograph showing the cross-sectional morphology of the Al layer synthesized by PLD. (**b**) Combined EDS elemental map illustrating the spatial distribution of all detected elements within the region shown in (**a**). (**c**–**f**) Individual EDS elemental maps revealing the distribution of (**c**) O, (**d**) Al, (**e**) Pt, and (**f**) Si, respectively.

The multilayer architecture of the Al/Pt/Si system was validated via cross-sectional SEM and EDS mapping. As illustrated in Figure 8, the Al thin film (~145 nm) exhibits a uniform morphology, overlaid upon a 160 nm Pt interlayer on the Si substrate. Elemental distribution maps confirm a cohesive Al layer capped by a thin, superficial Al_2_O_3_ passivation film. Consistent Pt and Si signals delineate the interlayer and substrate, respectively; the attenuated Si intensity is attributed to the limited interaction volume of the electron beam at the prescribed accelerating voltage.

Microstructure Al deposited on the surface of Pt-coated Si and chemical maps revealed similarly that Al and O elements were uniformly dispersed in the whole studied area.

The combined elemental distribution maps presented in Figure 8c reveal that the Al signal (magenta) is predominantly localized within the thin-film region, which corresponds to the dark layer observed in the SEM micrograph (Figure 8a). Within this same region, the detection of O is attributed to the presence of a passive Al_2_O_3_ layer covering the Al film. Furthermore, signals from Pt and Si are also detected within the Al-defined area; this is attributed to the electron beam interaction volume. This teardrop-like interaction region extends from the surface to a depth of approximately 5 μm within the solid specimen, acting as the source of secondary electrons, backscattered electrons, and characteristic X-rays. Figure 8e,f display the individual maps for Pt and Si, respectively. As expected, the Si signal is concentrated in the substrate region, consistent with the system’s architecture where the Pt interlayer is situated between the Al-GNP film and the Si base. Cross-sectional SEM validates the continuous, uniform Al overlayer on Pt/Si, with homogeneous elemental maps underscoring multilayer integrity and superficial oxidation limited to O/Al ratios < 0.1 [1,10,26].

**Figure 9:**
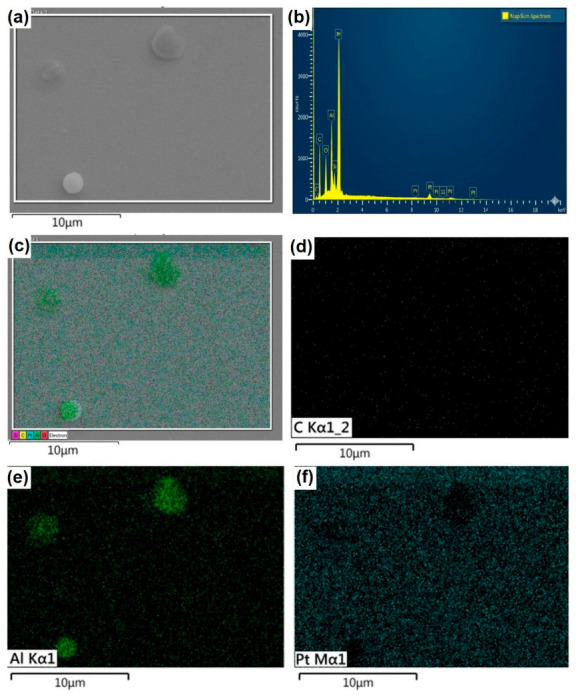
Surface characterization of the Al-GNP composite thin film deposited on a Pt-coated Si substrate. (**a**) SEM micrograph showing the surface morphology of the Al-GNP layer synthesized by PLD. (**b**) EDS microanalysis spectrum obtained from the region shown in (**a**). (**c**) Combined EDS elemental map illustrating the spatial distribution of all detected species within the analyzed area. (**d**–**f**) Individual EDS elemental maps revealing the distribution of (**d**) C, (**e**) Al, and (**f**) Pt, respectively.

Figure 9a illustrates the surface microstructure of the Al-GNP composite film, which uniformly covers the underlying substrate. The PLD process induced the formation of spherical micro-particles, clearly distributed across the film’s morphology. The corresponding EDS chemical spectra, acquired from the region shown in Figure 9a, are presented in Figure 9b, where C, O, Al, Pt, and Si are clearly identified. Although the carbon-derived signal is relatively weak—a behavior linked to the low concentration of graphene nanoplatelets within the Al matrix—this is further corroborated by the low-intensity C signal observed in the elemental map in Figure 9d [27,28].

The composite elemental map (Figure 9c) and individual Al signal (Figure 9e) corroborate a uniform dispersion across the analyzed surface. Notably, the intensified Al emission observed in the spherical particulates is attributed to local topographical effects inherent to the PLD process, rather than compositional gradients. Furthermore, the Pt signal (Figure 9f) confirms a continuous and homogeneous interlayer beneath the Al-GNP film, validating the structural integrity of the multicomponent architecture [29,30].

**Figure 10:**
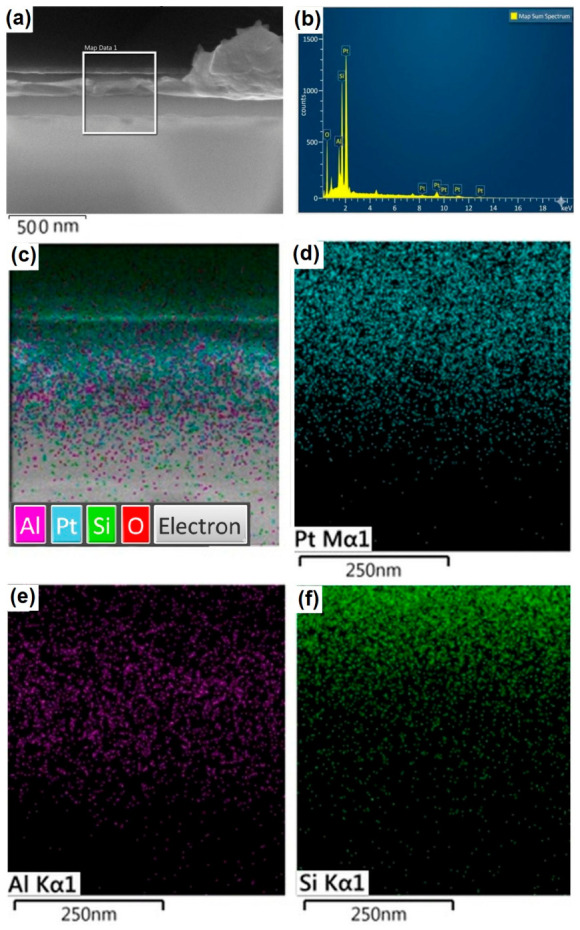
Cross-sectional microstructural and chemical characterization of the Al-GNP composite thin film on a Pt-coated Si substrate. (**a**) Cross-sectional SEM micrograph showing the morphology and layer thickness of the Al-GNP composite synthesized by PLD. (**b**) Representative EDS microanalysis spectrum acquired from the region indicated in (**a**), confirming the elemental purity. (**c**) Combined EDS elemental map illustrating the spatial distribution and interfacial integrity of all detected species (Al, C, Pt, Si, and O). (**d**–**f**) Individual EDS elemental maps revealing the distribution of (**d**) Pt, (**e**) Al, and (**f**) Si, respectively.

SEM-EDS characterization confirms the multilayer architecture of the Al-GNP/Pt/Si system. Cross-sectional SEM micrographs (Figure 10a) reveal a continuous Al thin film with a uniform thickness of ~122 nm, deposited over a ~160 nm Pt interlayer on the Si substrate. Elemental mapping (Figure 10c–f) demonstrates a homogeneous distribution of Al, O, Pt, and Si throughout the heterostructure. The Al and O signals correspond to the top Al layer, where the oxygen presence is attributed to a native surface Al_2_O_3_ passivating layer. Conversely, the Pt and Si signals confirm the integrity of the underlying interlayer and substrate, respectively.

The EDS point analysis (Figure 10b) further corroborates the elemental composition of the cross-section. The overlay map (Figure 10c) shows the Al signal (magenta) localized within the dark contrast region of the SEM micrograph. Although Pt and Si are detected within the upper layers, this is attributed to the electron beam interaction volume, which, for the acceleration voltages used, typically extends into a micrometer-scale droplet-shaped interaction region, generating characteristic X-rays from the underlying interfaces. As expected, the Si signal is concentrated in the bulk region, consistent with the Al-GNP/Pt/Si stack architecture.

Elemental mapping (Figure 10c–f) demonstrates homogeneous distributions of Al, O, Pt, and Si, where Al and O signals localize to the top layer—attributed to a native Al_2_O_3_ passivation (~2–5 nm)—while Pt and Si confirm the underlying interlayer and substrate integrity, respectively, despite minor overlap from the micrometer-scale subsurface interaction zone electron beam interaction volume at typical acceleration voltages (5–15 kV) [13,14,15,16,17,18,19,20,21,22,23,24,25,26,27,28,29,30,31]. The absence of detectable carbon (C) by EDS in Figure 10, may be attributed to the limited interaction volume between the electron beam and the cross-section of the deposited thin film. In this configuration, the beam interacts with a smaller sample volume due to the reduced thickness of the Al layer (approximately 122 nm). In contrast, EDS analyses performed on the film surface involve a larger interaction volume, increasing the likelihood of detecting carbon associated with the GNPs.

The uniform ~145 nm Al-GNP overlayer, void-free interfaces, and superficial O (3–8 at.%) precisely match PLD-reinforced Al films reported in 2019–2026 literature, where stoichiometric hybrids on Si/Pt exhibit GNP particulates amid Al droplets, columnar alignment, and sharp C/Al/O gradients without substrate interdiffusion, enhancing fracture strength via uniform GNP dispersion (1–5 μm lateral, 5–20 nm thick). Absence of agglomeration or carbide overgrowth contrasts sputtering’s dense Al-GNP matrices (20–80 nm grains, C/Al 0.5–1.5 wt%, O < 4 at%) yet echoes wafer-scale PLD’s low-defect C signals in Pt/Al-graphene stacks for CMOS compatibility. Pt/Si overlap in thin overlayers (<300 nm) is a standard EDS artifact, with reduced Si intensity reflecting Al-GNP attenuation akin to BSE contrasts in multilayers [6,32].

### 3.3. Surface Characterization via Atomic Force Microscopy (AFM) of Al-Based Thin Films

**Figure 11:**
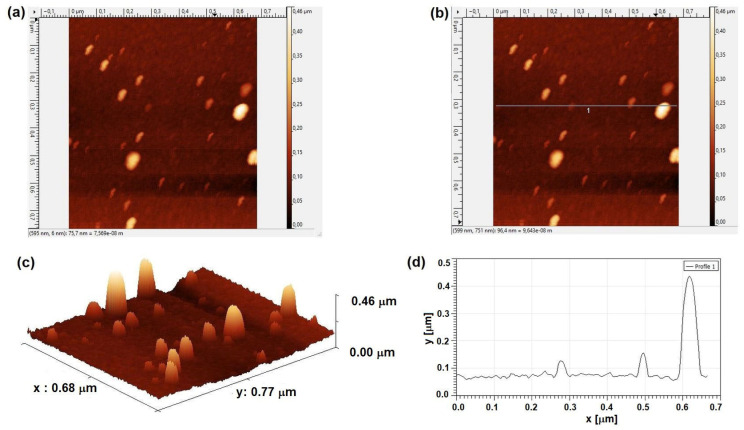
AFM surface characterization of the deposited Al layer: (**a**) 2D topographic micrograph (top view); (**b**) detailed scan area indicating the cross-sectional line for height profiling; (**c**) 3D surface reconstruction; and (**d**) topographic profile acquired along a 0.7 μm scan line.

Surface Morphology. As revealed by AFM micrographs (Figure 11a,d), the surface exhibits a dispersion of particles with rounded apices protruding from the Al base layer. These findings are consistent with scanning electron microscopy (SEM) observations, which show a relatively constant layer thickness.

The surface topography of the pure Al thin film reveals a high-amplitude roughness profile dominated by sharp features (Table 2). The RMS roughness (Sq = 35.4 nm) is significantly higher than the mean roughness (Sa = 19.1 nm), indicating a surface with substantial vertical deviations. The statistical distribution parameters show a high positive skewness (Ssk = 4.6), characterizing a peak-dominated surface where the morphology is primarily composed of protruding features rather than deep pits.

Furthermore, the extreme excess kurtosis reported (Sku = 31.1) identifies a leptokurtic distribution, confirming the presence of highly acute or “spiky” peaks. This specific morphology is consistent with the Volmer-Weber growth mode, where high-energy adatoms during the PLD process favor 3D island nucleation over layer-by-layer growth. The marked difference between the maximum peak height (Sp = 369 nm) and the maximum pit depth (Sv = 89.4 nm) further supports the conclusion of an outward-growing granular structure, which typically correlates with increased surface friction and mechanical interlocking potential.

Topographic profile analysis. The height profile acquired along a 0.7 μm scan line (Figure 11d) reveals three particles with rounded apices. Dimensional analysis of these features yielded widths of 43.3 nm, 32.5 nm, and 67.9 nm, with corresponding heights of 59 nm, 90.1 nm, and 360.6 nm, respectively. These dimensions confirm the nanometric scale of the observed features, which exhibit a pronounced vertical aspect ratio.

**Figure 12:**
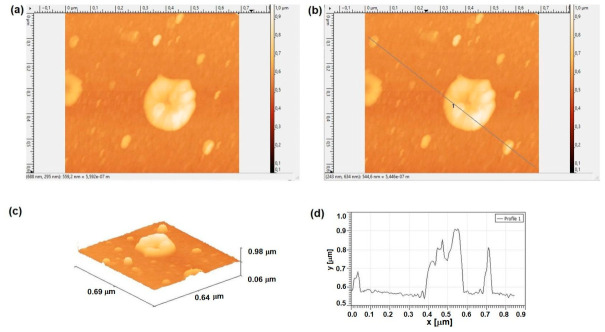
AFM surface characterization of the deposited Al-GNP layer: (**a**) 2D topographic micrograph (top view); (**b**) detailed scan area indicating the cross-sectional line for height profiling; (**c**) 3D surface reconstruction; and (**d**) topographic profile acquired along a 0.85 μm scan line.

Morphological analysis via AFM (Figure 12a–d) highlights a surface populated by distinct, round-topped particles that emerge from the underlying aluminum matrix. This topographical arrangement aligns well with our SEM cross-sectional data, which indicate that despite these surface features, the overall thickness of the deposited Al layer remains remarkably uniform.

Figure 12 presents the surface roughness parameters quantified by atomic force microscopy (AFM) for the aluminum thin film modified with graphene nanoplatelets (Al-GNP) deposited onto a Si/Pt substrate, revealing a nanometric heterogeneous morphology with prominent height variations (Sz = 920.7 nm).

The average height stands at 589 nm, suggesting a surface notably raised above the base substrate—a feature consistent with PLD deposition, which tends to produce rough topographies from particle coalescence and grain formation in Al-GNP composites (typically around 500–1000 nm median height) [33].

RMS roughness (Sq = 59.8 nm) and mean roughness (Sa = 35.4 nm) indicate a moderately rough texture, higher than that of pure quasiepitaxial Al films on Si (Sq ~ 6 nm), yet typical for GNP-reinforced systems where nanoplatelet dispersion increases graininess and interfacial exposure (Sq ranging 10–60 nm in reported studies) [34,35].

The marked positive skewness (Ssk = 2.8) points to a dominance of elevated peaks over valleys, likely due to exposed GNP protrusions or surface agglomerates within the Al matrix—features that could enhance interfacial scattering or tribological performance. The extreme excess kurtosis (Sku = 8.8, well above 3) underscores a height distribution with sharp peaks and pronounced outliers (Sp = 393.7 nm, Sv = 527 nm), characteristic of a “spiky” morphology arising from GNP-Al nanoheterogeneities, in stark contrast to smooth Gaussian surfaces (Sku ≈ 3) [36].

Overall, these metrics (Sq/Sa ≈ 1.7, average Sz ~ 900 nm) point to a robust Si-Pt/Al-GNP interface shaped by GNP loading, making the system promising for flexible electronics or field emission applications, where controlled ~ 50 nm textures boost conductivity while avoiding pinholes.

AFM analyses of pure Al and Al-GNP thin films on Si/Pt reveal similar rounded particle protrusions from the base layer—consistent with SEM uniform thickness and PLD-driven Volmer-Weber nucleation. Yet Al-GNP shows elevated roughness (Sq 59.8 nm, Sa 35.4 nm; Sz 920.7 nm) versus pure Al (Sq 35.4 nm, Sa 19.2 nm), a ~40–70% rise from GNP dispersion promoting 3D clustering and edge exposure, as in GNP-Al hybrids (Rq 10–64 nm) [37,38].

Both exhibit positive skewness (Ssk 4.68 > 2.84), marking peak-dominated topography, but GNP moderates extremes by filling interstices. Kurtosis contrasts sharply: pure Al’s leptokurtic spike (Sku 31.17; Sp/Sv 369/89 nm) signals sparse high-aspect islands, while Al-GNP’s milder tail (Sku 8.85; Sv 527 nm) reflects broader GNP-induced heterogeneities [39].

These shifts—higher amplitude, tempered spikiness—enhance interlocking for electronics/tribology (~50 nm optimal conductivity) and outperform pure Al in defect mitigation, favoring scalable flexible devices [40].

### 3.4. Microhardness of the Consolidated Al–GNP Composite

The microhardness results indicate that sintered pure Al reached 42.5 ± 2.7 HV. Once GNPs were introduced into the matrix, a clear improvement in hardness was observed. In particular, the Al–0.25 vol.% GNP composite achieved 121 ± 12.2 HV, which represents a notable increase compared with unreinforced aluminum. The composite also showed a wider scatter in hardness values than pure sintered Al. This behavior is likely related to the greater microstructural heterogeneity introduced by the graphene nanoplatelets, whereas the unreinforced aluminum exhibited a more uniform and consistent microstructure.

## 4. Conclusions

Through this study, Al-GNP composite thin films were successfully engineered from recycled aluminum sources, achieving an effective integration of sustainable powder consolidation process and Pulsed Laser Deposition (PLD) technology. Based on microstructural and experimental evidence, the following fundamental conclusions are drawn:

- Phase Integration and Structural Stability: The strategic dual-dispersion protocol—combining ultrasonication with magnetic stirring—proved to be a non-destructive and highly efficient route for ensuring the homogeneous integration of graphene nanoplatelets within the aluminum matrix. HR-TEM and XRD assessments confirmed that the structural integrity of the multilayer GNPs remained unaltered following the sintering and laser ablation stages. The presence of Al_4_C_3_ was not detected by the microstructural characterization techniques employed in this study, namely SEM, TEM, and XRD. However, its formation cannot be completely ruled out, as it could potentially be identified using higher-resolution techniques such as HRTEM. It is worth noting that the presence of this carbide may adversely affect the mechanical integrity of the Al–GNP interface.
- Sustainability and Target Synthesis: The transformation of aluminum from recycled beverage cans into high-purity targets validates a circular economy framework applicable to microelectronic-grade materials. The consolidated composite targets achieved high densities and phase purities that faithfully replicate the FCC structure of the α-Al phase, with no evidence of residual oxidation or contamination during the thermal cycles.
- GNP-Induced Morphological Regulation: Surface metrology revealed a profound shift in the film growth dynamics. While pure Al films exhibited a highly “spiky” and leptokurtic topography (Sku = 31.17)—characteristic of an unrestricted Volmer-Weber growth mode—the incorporation of 0.25 vol.% GNPs acted as a morphological regulator.

The composite films displayed a stabilized surface with a drastic reduction in excess kurtosis (Sku = 8.85), indicating that the graphene nanoplatelets effectively impede anisotropic vertical grain growth, favoring a morphology with blunter and more uniform apices.

- Interfacial and Stoichiometric Fidelity: Cross-sectional SEM-EDS characterization validated the precision of the PLD technique in transferring the composite stoichiometry to the substrate. PLD produced continuous, uniform pure Al and Al-GNP thin films with thicknesses of 145 and 122 nm, respectively. The stability of the carbon signal and the minimal oxygen content (3–8 at.%) underscore the viability of this architecture for precision applications under real operating conditions.

In summary, the Al-GNP/Pt/Si architecture developed in this work offers a promising pathway for next-generation microelectronic interconnects. The ability to tune surface roughness and microstructural evolution through graphene reinforcement provides a robust technical solution to the historical challenges of thermal fatigue and hillock formation in pure aluminum conductors.

Beyond conventional microelectronic interconnects, the obtained Al–GNP thin films may also be considered promising for broader advanced applications, including microelectronic packaging, diffusion barrier layers, thermal management coatings, wear-resistant conductive surfaces, MEMS/NEMS metallization, EMI shielding, sensor platforms, and corrosion-mitigating coatings. These potential uses are supported by the combined advantages of improved interfacial integrity, controlled morphology, lightweight metallic character, and the multifunctional properties associated with graphene-based reinforcements. Therefore, the present results position these films as attractive candidates for interfacial engineering and multifunctional thin-film technologies, rather than exclusively as immediate interconnect replacements.

Future work should include electrical resistivity mapping, thermal cycling reliability tests, and broader statistical surface morphology analyses to further validate the technological performance and application readiness of the Al–GNP thin films.

## Figures and Tables

**Table 1:** Typical Chemical Composition of Aluminum Used in Beverage Cans.

Element	Typical Range (wt.%)	Role/Origin
Al	Balance (96.0–99.0)	Main matrix element
Mn	0.8–1.5	Strengthening, formability
Mg	0.8–1.3	Solid solution strengthening
Fe	0.2–0.8	Residual impurity/recycled feedstock
Si	0.1–0.4	Residual impurity
Cu	0.05–0.25	Residual/alloying contribution
Zn	0.01–0.25	Trace residual element
Ti	0.01–0.10	Grain refinement
Cr	0.00–0.05	Trace alloying element
Others (each)	<0.05	Minor residuals
Others (total)	<0.15	Combined trace content
Al	Balance (96.0–99.0)	Main matrix element
Mn	0.8–1.5	Strengthening, formability
Mg	0.8–1.3	Solid solution strengthening

**Table 2:** Surface roughness parameters of pure Al and Al-0.25 vol.% GNP composite thin films deposited by PLD onto Pt/Si substrates.

Surface Roughness Property	Value	Value
Average value	89.4 nm	588.9 nm
RMS roughness (Sq)	35.4 nm	59.8 nm
RMS (grain-wise)	35.4 nm	59.8 nm
Mean roughness (Sa)	19.1 nm	35.4 nm
Asimetría (Ssk)	4.6	2.8
Excess kurtosis	31.1	8.8
Minimum	0.0 nm	61.9 nm
Maximum	458.4 nm	982.6 nm
Mode	83.1 nm	572.5 nm
Maximum peak height (Sp)	369 nm	393.7 nm
Maximum pit depth (Sv)	89.4 nm	527 nm
Maximum height (Sz)	458.4 nm	920.7 nm

## Data Availability

For availability of data from this study, please contact the corresponding author.
